# Shoulder Pain in a 33-Year-Old Male Found to Have Multiple Myeloma

**DOI:** 10.7759/cureus.107152

**Published:** 2026-04-16

**Authors:** Edward Eusanio, Rida Altaf, Hatem Hassanein, Steve Carlan

**Affiliations:** 1 Department of Internal Medicine, Orlando Regional Medical Center, Orlando, USA; 2 Department of Hematology and Medical Oncology, Orlando Health Cancer Institute, Orlando, USA; 3 Department of Academic Affairs and Research, Orlando Regional Medical Center, Orlando, USA

**Keywords:** autologous hematopoietic stem cell transplant, daratumumab, multiple myeloma exclusion, multiple myeloma treatment, pelvic malignancies irradiation

## Abstract

Multiple myeloma (MM) is a blood cancer involving monoclonal plasma cells, mainly diagnosed in older adults. Only a very small percentage of patients are diagnosed before age 40.

A 33-year-old male presented with hip and shoulder pain and was diagnosed with Immunoglobulin G (IgG) kappa multiple myeloma. Of the CRAB (calcium elevation, renal insufficiency, anemia, and bone lesions) criteria, he only exhibited anemia and bone lesions. He was found to have extensive osseous lesions throughout his skeleton and a bone marrow biopsy showing plasma cell myeloma involving 30% of medullary cellularity. He was started on therapy with daratumumab, lenalidomide, bortezomib, and dexamethasone, along with palliative radiation. Four months after his diagnosis, he underwent a successful autologous hematopoietic stem cell transplant, with recovery of his cell lines and improvement in his lab indices.

MM frequently exhibits non-specific symptoms like anemia, bone pain, fatigue, weight loss, and renal issues. This can cause it to be overlooked, resulting in delays in diagnosis and treatment, which increase morbidity and mortality. The challenge is greater when MM appears in younger patients, as its rarity leads to lower consideration. Physicians must maintain a high level of suspicion to diagnose and treat MM promptly, particularly in younger groups.

## Introduction

Multiple myeloma (MM) is a hematological malignancy characterized by the monoclonal proliferation of plasma cells. It is primarily diagnosed in older individuals, with the median age at diagnosis being 69 years [1]. Incidence data in the United States from 2000-2020 indicate that less than 15% of cases were diagnosed in patients under 55 years old [2]. MM is even rarer in younger individuals, with only 2% of patients under 40 years old at diagnosis [3]. The incidence of MM has been increasing in the United States over recent decades and accounts for 2% of cancer-related deaths [4]. The presence of signs from the CRAB criteria (hypercalcemia, renal impairment, anemia, and bone disease) often prompts clinicians to consider multiple myeloma as a potential diagnosis. Although the initial signs and symptoms are often nonspecific and include anemia (73%), bone pain (58%), elevated creatinine (48%), fatigue (32%), hypercalcemia (28%), and weight loss (24%) [3], the combination of these nonspecific symptoms and rarity of MM in younger individuals can lead to it being overlooked, resulting in delayed diagnosis and increased morbidity and mortality [5].

The pathophysiology of the CRAB criteria stems from the monoclonal proliferation of plasma cells. Plasma cells are responsible for the production of antibodies, and the plasma cells in MM produce monoclonal antibodies. The monoclonal production of plasma cells causes damage to the bone marrow, resulting in cytopenias. Monoclonal antibodies accumulate and precipitate in urine (also known as Bence-Jones proteins), which can lead to renal failure. Additionally, the activation of osteoclasts results in the lytic destruction of bone, causing pain and hypercalcemia [4]. This case describes a 33-year-old male presenting with shoulder and hip pain who was ultimately diagnosed with MM.

## Case presentation

The patient is a 33-year-old male from Haiti with no significant past medical history who came to the emergency department complaining of right shoulder and hip pain lasting for two weeks. Physical exam showed tenderness on the right anterior chest wall, tenderness on the right hip flexor, and pain with range of motion of the right hip. X-rays of the right shoulder and hip showed no signs of an acute process. No laboratory tests were done during this visit. The patient was discharged with methocarbamol. Two months later, he returned with the same symptoms, now along with new-onset fatigue. He denied other symptoms such as night sweats or weight loss. He also denied tobacco, alcohol, or drug use. Physical exam revealed a bony protrusion felt on the right clavicle and tenderness to palpation of the right iliacus.

Labs showed a hemoglobin of 11.0 g/dL (grams/deciliter) (reference range 12.6-16.6 g/dL), a slightly low serum albumin of 3.2 g/dL (reference range 3.5-5.0 g/dL), a high total serum protein of 10.0 g/dL (reference range 6.4-8.3 g/dL), and a high sedimentation rate of 107 mm/hr (reference range 0-15 mm/hr). Due to the patient's anemia, bony lesion, and elevated globulin gap, electrophoresis, immunoglobulin levels, and immunofixation were ordered to evaluate for MM. Serum protein electrophoresis demonstrated an M spike of 3.7 g/dL, and immunofixation showed monoclonal IgG kappa immunoglobulin. IgG, gamma globulin, kappa free light chain, and kappa/lambda free light chain ratio were all elevated as shown in Table 1.

**Table 1:** Lab values from serum protein electrophoresis and immunofixation at the time of diagnosis, after induction with D-RVd, and post-transplant. g/dL = grams per deciliter; mg/dL = milligrams/deciliter.

Lab	Reference Range	At Diagnosis	After D-RVd, before transplant	Post-Transplant
M (monoclonal) Spike	0 g/dL	3.7 g/dL	0.4 g/dL	0.2 g/dL
Gamma Globulin	0.5 g/dL to 1.6 g/dL	4.0 g/dL	0.7 g/dL	0.5 g/dL
IgG (Immunoglobulin G)	751 mg/dL to 1,560 mg/dL	5,241 mg/dL	703 mg/dL	405 mg/dL
IgA (Immunoglobulin A)	82 mg/dL to 453 mg/dL	35 mg/dL	11 mg/dL	8 mg/dL
IgM (Immunoglobulin M)	46 mg/dL to 304 mg/dL	22 mg/dL	14 mg/dL	7 mg/dL
Kappa Free Light Chain	0.33 mg/dL to 1.94 mg/dL	5.22 mg/dL	0.3778 mg/dL	0.1481 mg/dL
Lambda Free Light Chain	0.5700 mg/dL to 2.63 mg/dL	0.6479 mg/dL	0.2239 mg/dL	0.1994 mg/dL
Kappa/Lambda Free Light Chain Ratio	0.26 to 1.65	8.06	1.69	0.7427

**Figure 1:**
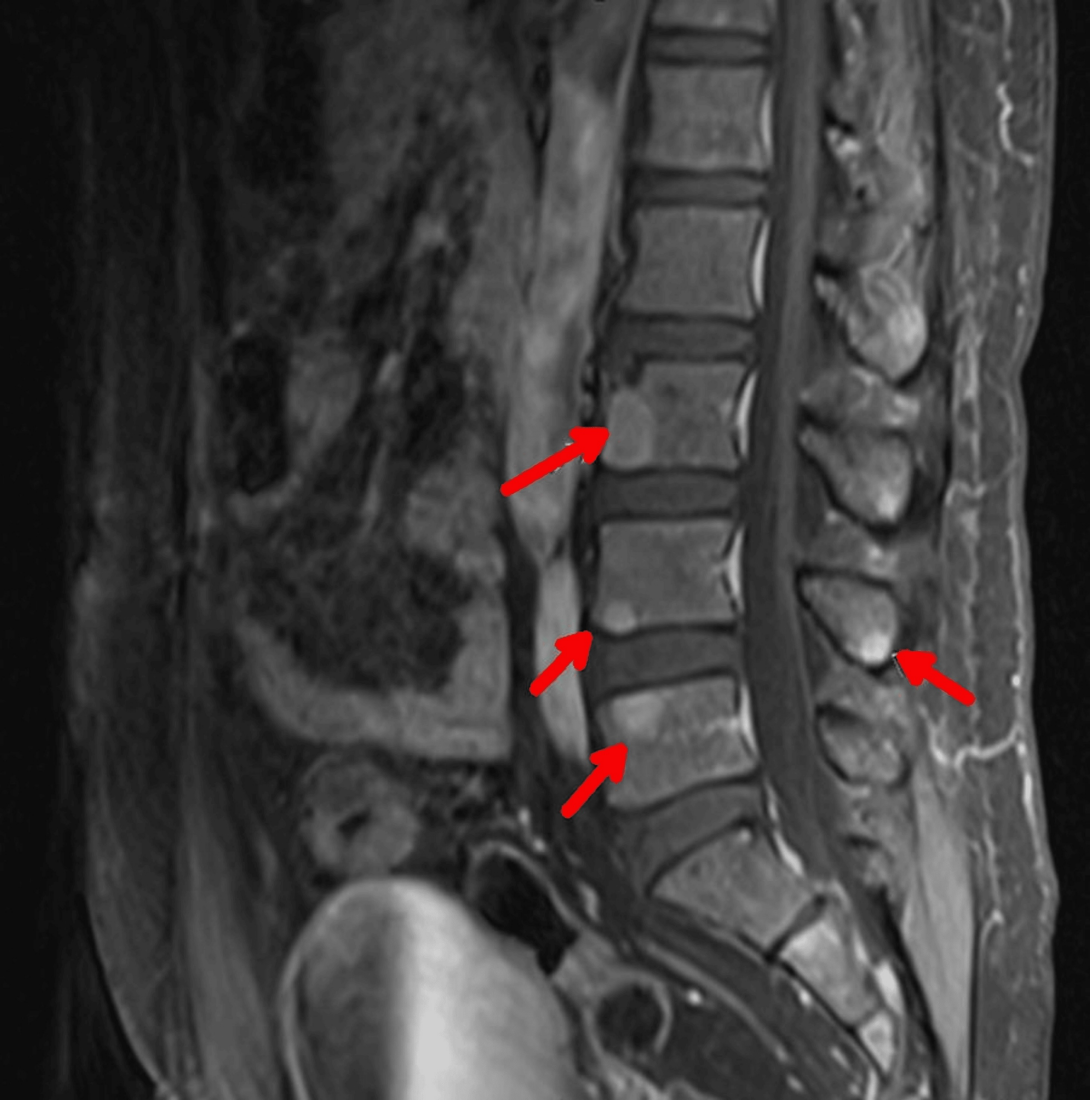
MRI (magnetic resonance imaging) lumbar spine with contrast showing widespread osseous enhancing lesions (red arrows). Osseous lesions can be seen in many conditions, but in conjunction with the patient’s initial lab values they are concerning for MM.

Beta-2 microglobulin was not obtained at the time of diagnosis. A CT (computed tomography) myeloma scan revealed multiple lytic lesions throughout the cervical spine, chest, abdomen, pelvis, and bilateral lower extremities. MRI (magnetic resonance imaging) of the entire spine and pelvis with contrast also identified the same lesions seen in the CT (Figure 1).

The patient underwent a bone marrow biopsy, which revealed 40% cellular marrow with trilineage hematopoiesis and 30% involvement by plasma cell myeloma. Flow cytometry of the bone marrow aspirate showed a monoclonal plasma cell population with kappa chain restriction. FISH analysis indicated monosomy 13, duplication of 1q, atypical t(4;14), and loss of MAF or chromosome 16/16q. Cytogenetics were normal.

**Figure 2:**
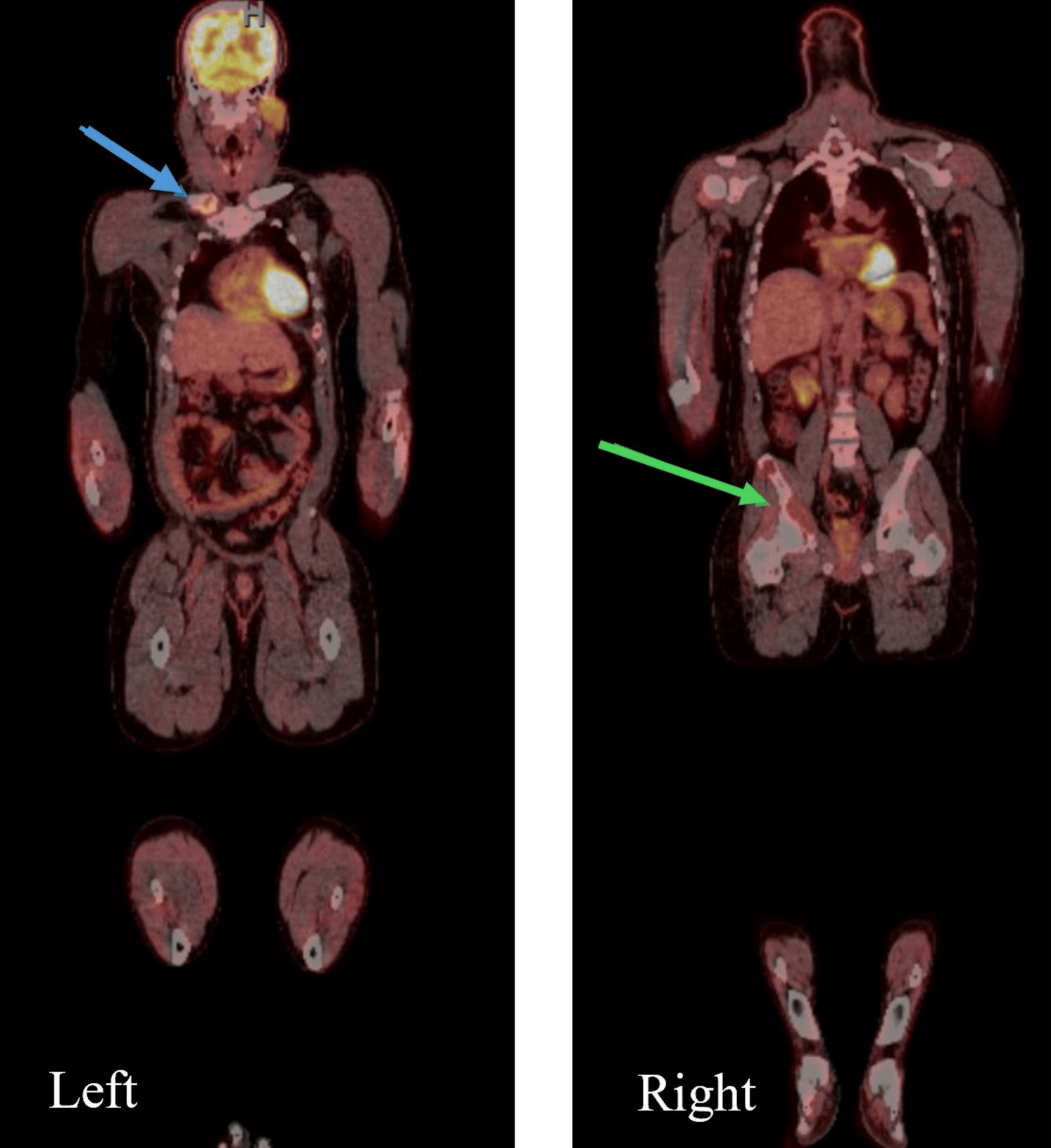
Total body PET/CT (Positron Emission Tomography / Computed Tomography) scan showing known widespread lesions with increased fluorodeoxyglucose uptake in the head of the right clavicle (left image, blue arrow) and right iliac bone and acetabulum (right image, green arrow), indicating areas with increased metabolic activity.

He was referred to hematology and started induction therapy with daratumumab, lenalidomide, bortezomib, and dexamethasone (D-RVd), along with palliative radiation to his pelvis. Three months after his diagnosis, he was in partial remission of his multiple myeloma, as shown by improved lab indices in Table 1. A repeat bone marrow biopsy at that time was negative for plasma cell neoplasm, with 40% cellular marrow. PET/CT (Positron Emission Tomography / Computed Tomography) also at that time revealed known widespread lucent lesions without extraosseous disease (Figure 2).

Four months after his diagnosis, he successfully underwent an autologous hematopoietic stem cell transplant, resulting in the complete recovery of his cell lines. He continues to receive maintenance therapy with lenalidomide and daratumumab. A bone marrow biopsy two months after the transplant was negative for plasma cell neoplasm, with 50% cellular marrow. He remains in a very good partial response (VGPR). Patient consent was obtained.

## Discussion

MM can be a difficult diagnosis to make in a timely manner due to several factors. Its nonspecific symptoms and lab findings are often mistaken for more common conditions, such as attributing renal impairment to longstanding hypertension [5]. A contributing factor is that patients typically first visit a general practitioner, who may not frequently encounter MM, with a median of three visits before referral to a hematologist [5,6]. In one study, 40% of MM patients experienced delays exceeding six months, leading to more complications and decreased disease-free survival, though overall survival was not significantly affected [7]. The nonspecific signs and symptoms also complicate ordering advanced tests like serum protein electrophoresis and making specialist referrals [5]. These challenges are especially evident in younger patients, given MM’s rarity in this age group [1,3]. A case report described a young patient with previous radiolucencies on X-rays who was diagnosed with MM only after suffering a pathological fracture several years later [8]. Another case involved a 27-year-old male diagnosed with light chain myeloma after a year of back pain, worsened by a fall; by the time of diagnosis, he needed a walker and had a large sacral mass with extensive bone involvement [9]. These cases show that delayed diagnosis in young patients can lead to increased morbidity and reduced quality of life.

Patients with delayed diagnoses often experience more MM-related complications even if the diagnosis is postponed by three months after symptoms appear [5,7]. Common issues include anemia (54%), bone disease (45%), and renal impairment (36%), with longer delays increasing the risk of complications [7]. A diagnostic delay raises morbidity and mortality because patients need more aggressive initial treatments, and options may become limited if renal failure occurs. Additionally, it diminishes quality of life due to ongoing bone pain and leads to late-stage disease signs that consume more healthcare resources [5].

Younger patients are more likely to have the light chain subtype of myeloma and tend to present with less advanced disease, often classified as International Staging System 1 [10]. Management of multiple myeloma revolves around determining a patient’s eligibility for autologous hematopoietic stem cell transplantation and several options for triple or quadruple therapies, selecting among several options of triplet or quadruplet therapies, leading to either transplantation and/or maintenance therapy [4]. Younger individuals typically tolerate intensive treatments like autologous stem cell transplants better, resulting in deeper remissions. However, they still experience significantly higher mortality rates compared to the general population, leading to more years of life lost [10]. A study comparing patients aged 21 to 40 years with those aged 41 to 60 years with MM found that younger patients had better 5- and 10-year overall survival rates (83% vs. 67% and 56% vs. 39%, respectively) [11]. This survival advantage was also observed when comparing patients who underwent autologous transplantation and those classified as International Staging System Stage 1, although the differences in survival rates did not appear in more advanced MM stages [11]. Cytogenetics are also important for prognosis in MM. On FISH analysis, the patient had duplication of 1q and t(4;14), which are both high-risk cytogenic abnormalities and are predictors for poor prognosis [12].

## Conclusions

MM is a blood cancer with high rates of morbidity and mortality. It is primarily diagnosed in older adults but can also affect younger patients. The main signs and symptoms, including CRAB criteria, are often non-specific, which can lead to delays in diagnosis and worsen the outlook. This is particularly problematic in younger individuals, as MM might not be initially suspected when evaluating their symptoms or lab results.

This case emphasizes the importance for clinicians to maintain a broad differential diagnosis and recognize symptom and lab result patterns that could indicate MM, even in unlikely patient groups like this one. Patients with MM often first seek care from primary providers such as general practitioners, urgent care centers, or emergency rooms, where their initial symptoms may be attributed to more common causes. Prompt diagnosis and treatment are vital for better outcomes in these patients. Further research should be undertaken to further identify reasons why the diagnosis of MM is overlooked in younger patients and what interventions could be taken to better identify these patients to reduce diagnostic delays.
